# Independent and joint associations of glucose modified by high-density lipoprotein cholesterol with mortality in heart failure patients: evidence from the Jiangxi, China cohort

**DOI:** 10.3389/fendo.2025.1680746

**Published:** 2025-10-29

**Authors:** Guoan Jian, Shiming He, Kun Jiang, Zhenyu Wang, Juan Wang, Houhui Lan, Guobo Xie, Guotai Sheng, Yan Fang, Wei Wang, Yang Zou

**Affiliations:** ^1^ Jiangxi Medical College, Nanchang University, Nanchang, Jiangxi, China; ^2^ Jiangxi Cardiovascular Research Institute, Jiangxi Provincial People’s Hospital, The First Affiliated Hospital of Nanchang Medical College, Nanchang, Jiangxi, China; ^3^ Department of Cardiology, Jiangxi Provincial People’s Hospital, The First Affiliated Hospital of Nanchang Medical College, Nanchang, Jiangxi, China

**Keywords:** fasting plasma glucose, high-density lipoprotein cholesterol, fasting plasma glucose to high-density lipoprotein cholesterol ratio, acute decompensated heart failure, China

## Abstract

**Introduction:**

Metabolic disorders characterized by dysregulation of glucose and lipid homeostasis are significant drivers of heart failure progression. This study proposes using high-density lipoprotein cholesterol (HDL-C) to modify fasting plasma glucose (FPG), and thereby constructing the FPG/HDL-C ratio (FHR) as a novel comprehensive metabolic indicator, and further investigates the synergistic effects of the FHR and its components on mortality in patients with acute decompensated heart failure (ADHF).

**Methods:**

This cohort study included 2,328 ADHF patients recruited from the Jiangxi-ADHF II cohort. Multivariable Cox regression and restricted cubic spline models were used to analyze the association between the FHR and 30-day mortality in ADHF patients. To visualize the joint effects of FPG and HDL-C on 30-day mortality risk, we generated two-dimensional heatmaps and three-dimensional surface plots. Finally, mediation models were employed to perform exploratory analysis of the potential mediating roles of inflammatory factor white blood cells, oxidative stress marker gamma-glutamyl transferase, and nutritional factor albumin in the association between FHR and mortality risk.

**Results:**

During the 30-day follow-up, 150 deaths (6.44%) occurred in the Jiangxi-ADHF II cohort. Multivariable Cox regression analysis revealed a positive association between FHR and 30-day mortality. Restricted cubic spline analysis showed a U-shaped association instead of a linear pattern, with the lowest mortality risk at FHR values ranging from 4 to 6. Notably, the joint association analysis based on two-dimensional heatmaps and three-dimensional surface plots, demonstrated a concave-shaped association of FPG and HDL-C with 30-day mortality: when both FPG (3–7 mmol/L) and HDL-C (1.05–1.65 mmol/L) were maintained within specific ranges, short-term mortality risk was minimized. Finally, mediation analysis suggested that inflammatory factor white blood cells and the nutritional factor albumin play significant mediating roles in the short-term mortality risk of ADHF patients associated with FHR.

**Discussion:**

This cohort study of the Jiangxi population in China is the first to reveal a U-shaped association between FHR and 30-day mortality in ADHF patients, establishing a synergistic effect of FPG and HDL-C on mortality risk. Based on these findings, we propose implementing a “metabolic synergistic management” strategy for ADHF patients in clinical practice.

## Graphical Abstract

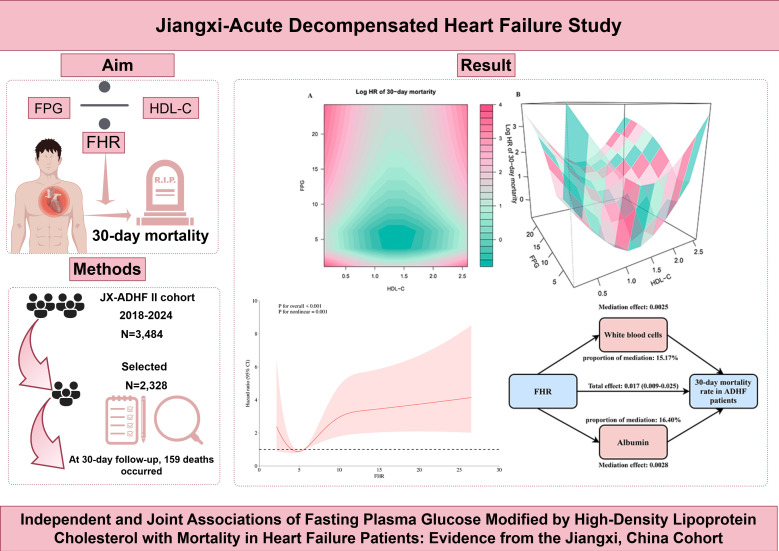

## Background

Heart failure (HF) is a terminal cardiovascular syndrome characterized by structural or functional cardiac abnormalities that result in impaired ventricular filling and reduced cardiac output (1). Epidemiological studies indicate that HF affects more than 50 million individuals globally, with the disease burden predominantly concentrated in low- and middle-income countries (2, 3). In China, the number of HF patients is approximately 12.1 million, accounting for one-fourth to one-fifth of the total number of cases worldwide. With population aging accelerating and metabolic diseases (e.g., hypertension, diabetes, dyslipidemia) becoming more prevalent, the incidence of HF is projected to continue rising in the future (4). Notably, HF has emerged as one of the leading causes of hospitalization among individuals aged 65 and older, with the vast majority of these admissions attributed to acute decompensated HF (ADHF) (1, 3). ADHF represents an acute and critical cardiovascular condition; despite significant advancements in treatment strategies in recent years, the incidence of short-term adverse outcomes remains persistently high in this population (5–7), imposing a substantial economic burden on both society and patients’ families.

As with other critical illnesses, the progression of ADHF is closely associated with metabolic disorders, with stress-induced hyperglycemia as one of its prominent manifestations (8, 9). Multiple large-scale cohort studies have demonstrated that nearly 50% of ADHF patients present with elevated glucose at admission (10, 11). As a core component of metabolic syndrome (12), hyperglycemia exacerbates cardiac injury through mechanisms including activating inflammatory pathways, enhancing oxidative stress, and aggravating insulin resistance (IR) (13–15). However, the association between hyperglycemia and short-term prognosis in ADHF remains controversial. While most studies report an independent positive association (9, 11, 16–18), others have found no significant correlation (10, 19, 20). This heterogeneity suggests that a single biomarker (e.g., glucose) may be inadequate to reflect the complexity of metabolic dysregulation in ADHF, thus limiting its prognostic value. Notably, high-density lipoprotein cholesterol (HDL-C), another key component of metabolic syndrome (12), plays multiple protective roles in the pathophysiology of ADHF: On one hand, HDL-C maintains glucose metabolic homeostasis by enhancing insulin sensitivity and improving β-cell function (21, 22); on the other hand, it exerts anti-ventricular remodeling effects and improves diastolic function through mechanisms such as inhibiting inflammatory cytokine release, counteracting oxidative stress, and suppressing cardiomyocyte apoptosis, thereby delaying the progression of HF (23–25). Considering the potential antagonistic effects of hyperglycemia and HDL-C, this study proposes the use of HDL-C to modify fasting plasma glucose (FPG), thereby constructing the FPG/HDL-C ratio (FHR) as a novel comprehensive metabolic indicator, and further analyzes the synergistic effects of the FHR and its components on mortality in patients with ADHF.

## Methods

### Study population

**Figure 1:**
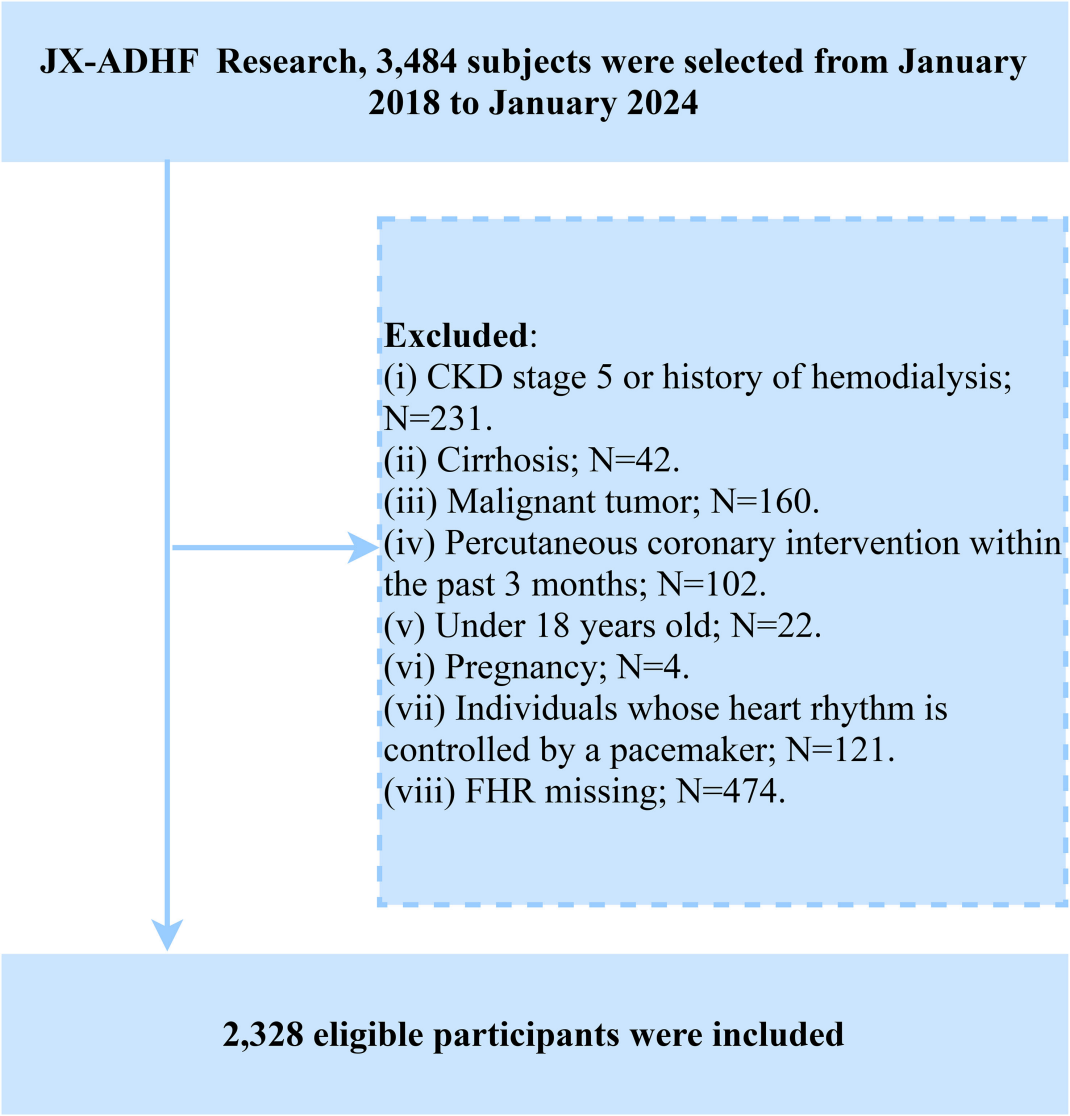
Flow chart for inclusion and exclusion of study participants.

The Jiangxi-ADHF II is a retrospective cohort study initiated by Jiangxi Provincial People’s Hospital. Based on clinical data collected during hospitalization and subsequent follow-up information of ADHF patients, this study aims to develop novel early risk stratification methods and provide evidence-based guidance for improving adverse prognostic outcomes in this patient population. The study consecutively enrolled 3,484 ADHF patients who were admitted to Jiangxi Provincial People’s Hospital between January 2018 and January 2024. The diagnostic criteria strictly adhered to the latest editions of the guidelines for HF management issued by the European Society of Cardiology and American College of Cardiology/American Heart Association during the study period. In the current study, we excluded patients with the following characteristics: (1) To minimize the additional impact of non-cardiac fluid and sodium retention, we excluded patients with diagnosed uremia and those receiving hemodialysis due to chronic kidney disease (n=231), as well as patients with liver cirrhosis (n=42). (2) To minimize the impact of malignancy on survival rates, we excluded patients with comorbid malignancies (n=160). (3) To reduce the influence of reperfusion therapy on short-term outcomes, we excluded patients who had undergone percutaneous coronary intervention within the past 3 months (n=102). (4) Patients with pacemakers were excluded due to potential autonomic nervous system dysfunction (n=121). (5) Minors (n=22) and pregnant participants (n=4) were excluded. (6) Patients with missing FHR data were excluded (n=474). Ultimately, a total of 2,328 patients were included in the study. The detailed inclusion/exclusion criteria flowchart is presented in **Figure 1**.

### Ethics approval

The study protocol was reviewed and approved by the Ethics Review Committee of Jiangxi Provincial People’s Hospital (IRB: 2024-001), and informed consent for data use was obtained from all participants or their legal guardians. The entire research process strictly adhered to the ethical principles for medical research outlined in the Declaration of Helsinki. The study reporting fully complied with the Strengthening the Reporting of Observational Studies in Epidemiology guidelines.

### Data collection

Demographic data (gender, age), clinical comorbidities (hypertension, diabetes, coronary heart disease [CHD], stroke), New York Heart Association (NYHA) functional classification, lifestyle factors (smoking, drinking status), and echocardiographic parameters (left ventricular ejection fraction [LVEF]) were collected by two rigorously trained researchers at the time of patient admission, with comorbidities ascertained through medical histories and medication records.

Blood samples were collected within 24 hours of patient admission and analyzed at the Clinical Laboratory Center of Jiangxi Provincial People’s Hospital. The measured parameters included hematological indices (red blood cell count [RBC], white blood cell count [WBC], platelet count [PLT]), biochemical markers (albumin [ALB], alanine aminotransferase [ALT], aspartate aminotransferase [AST], gamma-glutamyl transferase [GGT], creatinine [Cr], uric acid [UA]), lipid metabolism components (total cholesterol [TC], triglycerides [TG], low-density lipoprotein cholesterol [LDL-C], HDL-C), and other parameters (FPG, N-terminal pro-B-type natriuretic peptide [NT-proBNP]). Of particular note, blood samples for FPG, liver enzymes, and lipid metabolism parameters were strictly obtained under fasting conditions (requiring ≥8 hours of fasting either at admission or the following morning) to eliminate dietary interference and ensure the reliability of the laboratory results.

### Study outcomes

The primary outcome of this study was all-cause mortality occurring within 30 days after hospital admission. Using the admission date as the start of the follow-up, survival status was ascertained through follow-up by trained medical professionals via telephone calls, text messages, and in-person interviews in outpatient or inpatient settings.

### Handling of missing data

Data analysis indicated (**Supplementary Table 1**) that missingness proportions across study variables ranged from 0% to 4.47%, with a maximum of 104 missing cases. The missingness pattern plot (**Supplementary Figure 1**) revealed a strong correlation in missingness patterns among biochemical parameters (UA, Cr, ALT, AST, GGT, ALB) and hematological parameters (WBC, RBC, PLT), suggesting that these data might be missing at random. Furthermore, LVEF exhibited relative independence from other missing variables, suggesting its missingness might be completely at random. Given the low overall missingness rate and consistency with the missing at random assumption, this study analyzed the original dataset without imputation.

### Statistical analysis

Participants were categorized into quartiles (Q1-Q4) based on the quartiles of FHR values. Quantitative data were presented as mean ± standard deviation for normal distribution and median (interquartile range) for non-normal distribution, respectively. Categorical variables were expressed as counts (percentages). Between-group comparisons were performed using parametric tests (one-way ANOVA) and non-parametric tests (Kruskal-Wallis test) for continuous variables, while categorical variables were analyzed using the chi-square (χ²) test.

Kaplan-Meier analysis was performed to plot survival curves for ADHF patients across different FHR groups, and the log-rank test was applied to assess differences between groups. Three multivariable Cox proportional hazards regression models were subsequently constructed to quantify the strength of the association between FHR and 30-day mortality by calculating hazard ratios (HRs) and 95% confidence intervals: Model I adjusted for demographic characteristics and comorbidities (gender, age, hypertension, diabetes, stroke, CHD); Model II further adjusted for lifestyle factors and cardiac function (NYHA classification, drinking status, smoking status, LVEF) based on Model I; Model III additionally adjusted for laboratory parameters (WBC, RBC, PLT, AST, GGT, ALB, Cr, UA, TC, TG, LDL-C, and NT-proBNP) based on Model II adjustments. Notably, prior to regression analysis, we excluded covariates with potential collinearity by calculating variance inflation factors and verified the proportional hazards assumption by evaluating Schoenfeld residuals.

Restricted cubic spline (RCS) analysis was conducted to evaluate the nonlinear association between FHR and 30-day all-cause mortality in ADHF patients based on the final Model III. Furthermore, using Cox regression and OpenGL technology, we generated heatmaps and three-dimensional surface plots to visualize the joint association of FPG and HDL-C with 30-day all-cause mortality, while quantifying the changes in the risk gradient underlying their synergistic effects.

Stratified analysis was conducted to evaluate potential population differences across three domains: 1) demographic characteristics (stratified by age and gender); 2) cardiac function parameters (stratified by NYHA classification and LVEF values); and 3) comorbidity status (stratified by the presence/absence of hypertension, diabetes, stroke, or CHD). Furthermore, interaction effects between stratification variables and FHR were quantitatively assessed using likelihood ratio tests.

To evaluate the predictive value of FHR for 30-day all-cause mortality in ADHF patients, we performed ROC analysis and calculated the following metrics: 1) the area under the curve (AUC) for predictive performance; 2) the optimal cutoff value; and 3) the corresponding sensitivity and specificity. Furthermore, DeLong’s test was employed to statistically compare the AUC values between different predictive models.

An exploratory mediation model was employed to evaluate the potential mediating roles of oxidative stress, inflammation, and nutritional factors in the association between FHR and 30-day all-cause mortality in ADHF patients. Based on prior evidence, GGT was selected as a biomarker for oxidative stress (26), WBC count as a marker for inflammation (27), and ALB as an indicator of nutritional status (28). The mediating effects were quantified using the bootstrap method (n=1000), calculated as the ratio of the indirect effect to the total effect.

Several sensitivity analyses were conducted to verify the robustness of the results: (1) To assess the generalizability of the findings, this study conducted external validation by investigating the association between UHR and all-cause mortality among patients with congestive HF in an independent sample from the 1998–2018 U.S. (United States) National Health and Nutrition Examination Survey. (2) Considering that pharmacotherapy may influence study outcomes by affecting glucose or lipid levels, we further adjusted for in-hospital treatment factors (including medications such as insulin, selective sodium glucose cotransporter 2 (SGLT2) inhibitors, statins, or diuretics) in multivariable models to address this potential confounding effect. Additionally, we evaluated interaction effects between different medications (including insulin, SGLT2 inhibitors, statins, or diuretics) and FHR-related mortality outcomes. (3) Given that body mass index is a significant prognostic factor in HF patients, we further performed an exploratory sensitivity analysis to assess whether the association between FHR and mortality in ADHF patients remained consistent across obese and non-obese populations.

## Results

### Baseline characteristics of the study population stratified by FHR

This study included a total of 2,328 patients with ADHF, comprising 1,367 males (58.72%) and 961 females (41.28%), with a mean age of 69 years. Baseline characteristics grouped by FHR quartiles are presented in **Table 1**. Overall, compared to ADHF patients in Q1, those in Q4 were younger, with a higher proportion of males and NYHA Class IV, and a significantly elevated prevalence of diabetes, hypertension, and CHD. Regarding laboratory parameters, Q4 exhibited higher levels of WBC count, PLT count, ALT, AST, GGT, Cr, UA, TG, and NT-proBNP, but lower levels of ALB, TC, LDL-C, and HDL-C. However, no significant differences were observed in RBC count, smoking status, drinking status, and stroke prevalence across FHR groups.

**Table 1:** Summary of baseline characteristics of the study population according to FHR quartile group.

Variable	FHR quartiles				*P*-value
	Q1	Q2	Q3	Q4	
No. of subjects	582	581	581	584	
Age (years)	73.00 (64.00-81.00)	71.00 (59.00-80.00)	70.00 (61.00-79.00)	69.00 (58.75-78.00)	<0.001
Gender (n,%)					<0.001
Male	290 (49.83%)	335 (57.66%)	358 (61.62%)	384 (65.75%)	
Female	292 (50.17%)	246 (42.34%)	223 (38.38%)	200 (34.25%)	
Hypertension (n,%)				0.046
No	331 (56.87%)	335 (57.66%)	318 (54.73%)	293 (50.17%)	
Yes	251 (43.13%)	246 (42.34%)	263 (45.27%)	291 (49.83%)	
Diabetes (n,%)					<0.001
No	527 (90.55%)	489 (84.17%)	422 (72.63%)	265 (45.38%)	
Yes	55 (9.45%)	92 (15.83%)	159 (27.37%)	319 (54.62%)	
Stroke (n,%)					0.556
No	481 (82.65%)	490 (84.34%)	482 (82.96%)	474 (81.16%)	
Yes	101 (17.35%)	91 (15.66%)	99 (17.04%)	110 (18.84%)	
CHD (n,%)					<0.001
No	417 (71.65%)	403 (69.36%)	383 (65.92%)	349 (59.76%)	
Yes	165 (28.35%)	178 (30.64%)	198 (34.08%)	235 (40.24%)	
NYHA classification (n,%)				<0.001
III	435 (74.74%)	413 (71.08%)	371 (63.86%)	357 (61.13%)	
IV	147 (25.26%)	168 (28.92%)	210 (36.14%)	227 (38.87%)	
Smoking status (n,%)				0.707
No	486 (83.51%)	479 (82.44%)	473 (81.41%)	489 (83.73%)	
Yes	96 (16.49%)	102 (17.56%)	108 (18.59%)	95 (16.27%)	
Drinking status (n,%)				0.627
No	517 (88.83%)	525 (90.36%)	523 (90.02%)	532 (91.10%)	
Yes	65 (11.17%)	56 (9.64%)	58 (9.98%)	52 (8.90%)	
LVEF (%)	48.00 (38.00-57.00)	47.00 (37.00-56.00)	45.00 (36.00-56.00)	45.00 (35.00-55.00)	0.001
WBC (×10^9^/L)	5.80 (4.72-7.57)	5.89 (4.64-7.40)	6.20 (4.90-7.97)	7.10 (5.60-9.60)	<0.001
RBC (×10^12^/L)	4.07 (0.72)	4.09 (0.77)	4.05 (0.78)	4.07 (0.84)	0.784
PLT (×10^9^/L)	160.00 (126.00-202.00)	160.00 (123.75-205.25)	169.00 (128.50-218.00)	171.00 (130.00-226.00)	0.006
ALB (g/L)	36.83 (4.85)	35.75 (4.61)	35.24 (4.74)	34.07 (5.39)	<0.001
ALT (U/L)	20.00 (13.00-33.00)	20.00 (13.75-34.00)	22.00 (14.00-39.00)	25.00 (15.00-50.00)	<0.001
AST (U/L)	26.00 (20.00-36.00)	25.00 (20.00-35.00)	26.00 (20.00-39.00)	27.00 (19.00-47.00)	0.047
GGT (U/L)	38.00 (23.00-67.00)	42.00 (25.00-68.00)	43.00 (25.00-76.50)	46.00 (28.00-83.00)	0.001
Cr (umol/L)	83.00 (65.00-110.00)	87.00 (70.00-113.50)	91.00 (75.00-126.00)	103.50 (76.75-155.25)	<0.001
UA (umol/L)	400.00 (325.00-512.00)	414.50 (333.50-520.00)	442.00 (346.75-554.75)	466.00 (363.75-596.75)	<0.001
TG (mmol/L)	1.01 (0.79-1.31)	1.08 (0.82-1.42)	1.18 (0.89-1.61)	1.35 (1.03-1.86)	<0.001
TC (mmol/L)	4.14 (3.56-4.75)	3.76 (3.18-4.53)	3.59 (3.02-4.26)	3.37 (2.77-4.11)	<0.001
LDL-C (mmol/L)	2.29 (1.78-2.86)	2.25 (1.74-2.87)	2.22 (1.76-2.77)	2.12 (1.66-2.74)	0.003
NT-proBNP (pmol/L)	3264.50 (1621.00-5203.25)	3358.00 (1832.00-5949.00)	3879.00 (1882.00-6845.00)	4222.50 (1984.50-7224.25)	<0.001
30-day mortality (n,%)	18 (3.09%)	18 (3.10%)	36 (6.20%)	78 (13.36%)	<0.001

CHD, coronary heart disease; NYHA, New York Heart Association; LVEF, left ventricular ejection fraction; TG, triglyceride; TC, total cholesterol; LDL-C, low-density lipid cholesterol; Cr, creatinine; UA, uric acid; WBC, white blood cell count; RBC, red blood cell count; PLT, platelet count; ALT, alanine aminotransferase; AST, aspartate aminotransferase; GGT, gamma-glutamyl transferase; ALB, albumin; NT-proBNP, N-Terminal Pro-Brain Natriuretic Peptide; FHR, fasting plasma glucose to high-density lipoprotein cholesterol ratio.

### Follow-up outcomes

**Figure 2:**
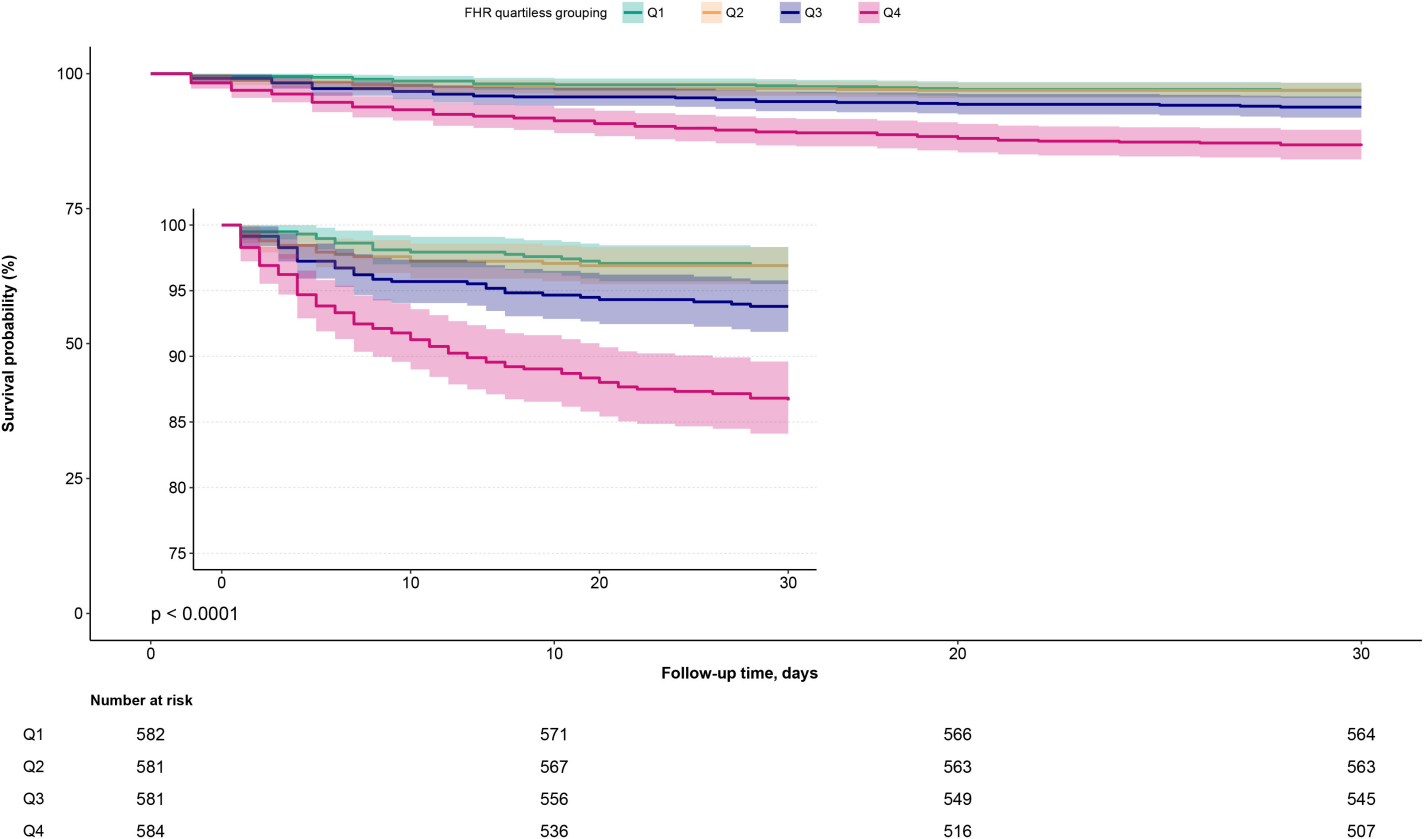
30-day survival curves of ADHF patients stratified by FHR quartiles. ADHF, acute decompensated heart failure; FHR, fasting plasma glucose to high-density lipoprotein cholesterol ratio.

During the 30-day follow-up, a total of 150 ADHF patients (6.44%) experienced mortality. **Figure 2** presents the 30-day survival curves of ADHF patients stratified by FHR, demonstrating that the Q4 group had a significantly higher 30-day mortality rate compared to the other three groups (Log-rank *p* < 0.0001).

### Association between FHR and 30-day mortality in ADHF patients

**Table 2** presents the HRs for the association between FHR (analyzed as both continuous and categorical variables) and 30-day mortality in ADHF patients. Despite progressive model adjustment, HRs progressively decreased while maintaining a consistent positive association overall. In the fully adjusted Model III, each 1-unit increase in FHR was associated with a 5% higher risk of 30-day mortality in ADHF patients (HR: 1.05, 1.02–1.08). Additionally, compared to the Q1 group, patients in Q4 had a 201% increased risk of 30-day mortality (HR: 3.01, 1.53–5.92). Across all models, FHR demonstrated a significant positive trend with 30-day mortality.

**Table 2:** Multivariable Cox regression analysis of the association between FHR and 30-day all-cause mortality in patients with ADHF.

Independent variable	HR (95%CI)			
	Non-adjusted	Model I	Model II	Model III
FHR	1.12 (1.10, 1.13)	1.12 (1.10, 1.14)	1.11 (1.08, 1.13)	1.05 (1.02, 1.08)
FHR (quartiles)				
Q1	1.0	1.0	1.0	1.0
Q2	1.01 (0.52, 1.94)	1.07 (0.55, 2.05)	0.99 (0.50, 1.94)	1.06 (0.53, 2.14)
Q3	2.04 (1.16, 3.59)	2.18 (1.23, 3.85)	1.77 (0.98, 3.19)	1.54 (0.81, 2.93)
Q4	4.55 (2.72, 7.59)	5.26 (3.06, 9.05)	4.14 (2.35, 7.31)	3.01 (1.53, 5.92)
*P*-trend	<0.01	<0.01	<0.01	<0.01

ADHF, acute decompensated heart failure; HR, hazard ratios; CI, Confidence interval; FHR, fasting plasma glucose to high-density lipoprotein cholesterol ratio.

Model I adjusted for: Gender, age, hypertension, diabetes, stroke, CHD;

Model II adjusted for: Gender, age, hypertension, diabetes, stroke, CHD, NYHA classification, dinking status, smoking status, and LVEF;

Model III adjusted for: Gender, age, hypertension, diabetes, stroke, CHD, NYHA classification, dinking status, smoking status, LVEF, WBC, RBC, PLT, AST, GGT, ALB, Cr, UA, TC, TG, LDL-C, NT-proBNP.

**Figure 3:**
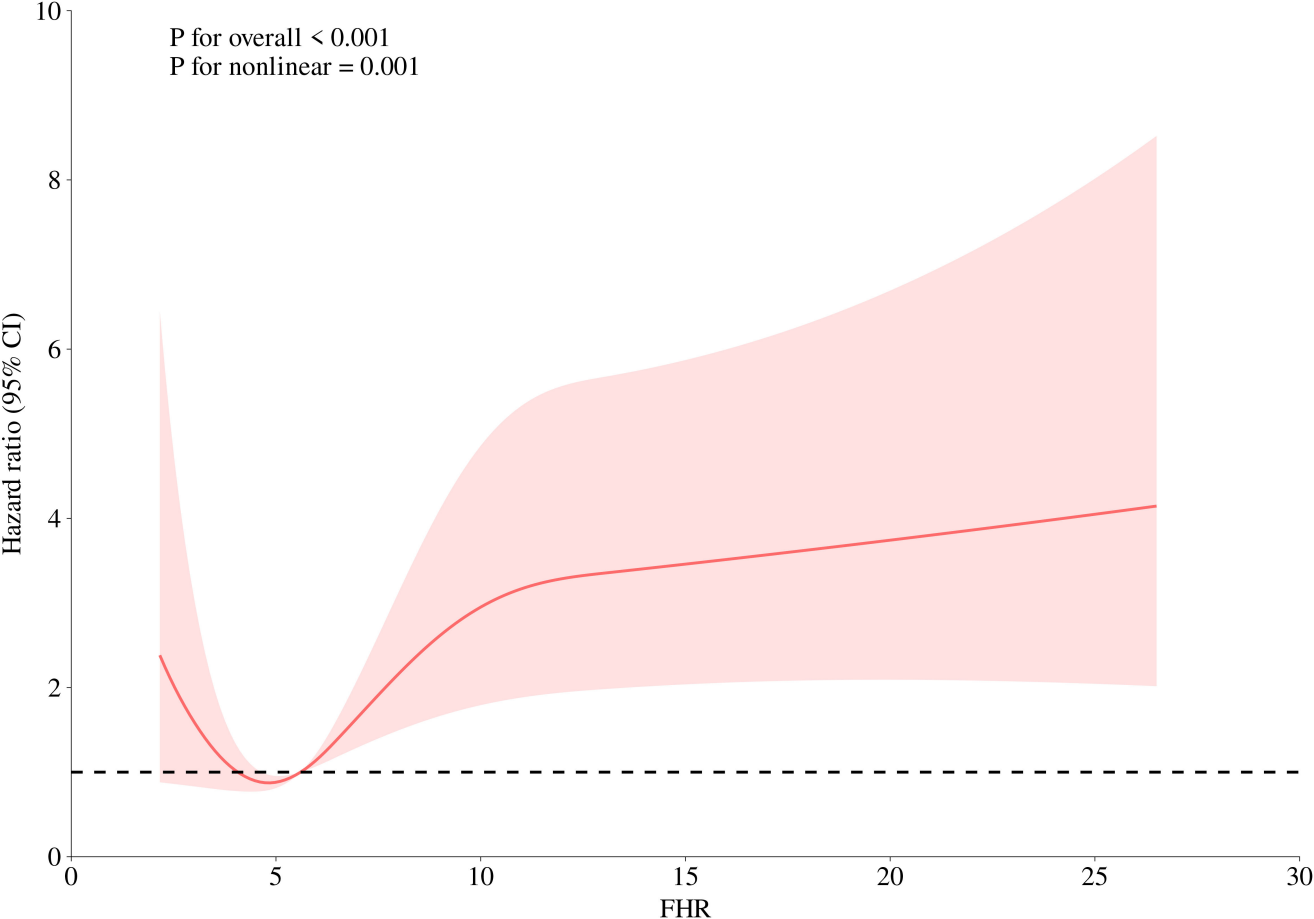
Fitting the dose-response relationship between FHR and 30-day all-cause mortality in ADHF patients with 4 knots restricted cubic spline. FHR, fasting plasma glucose to high-density lipoprotein cholesterol ratio; ADHF, acute decompensated heart failure. Adjusted for gender, age, hypertension, diabetes, stroke, CHD, NYHA classification, dinking status, smoking status, LVEF, WBC, RBC, PLT, AST, GGT, ALB, Cr, UA, TC, TG, LDL-C, NT-proBNP.

A 4-knot RCS was used to visualize the association pattern between FHR and short-term outcomes, revealing a significant non-linear positive association (**Figure 3**; *P* for non-linearity = 0.001). Specifically, the association between FHR and 30-day mortality in ADHF patients exhibited a U-shaped curve, with the lowest mortality risk observed when FHR ranged between 4–5 units.

### Joint association of FHR components (FPG and HDL-C) with 30-day mortality in ADHF patients

**Figure 4:**
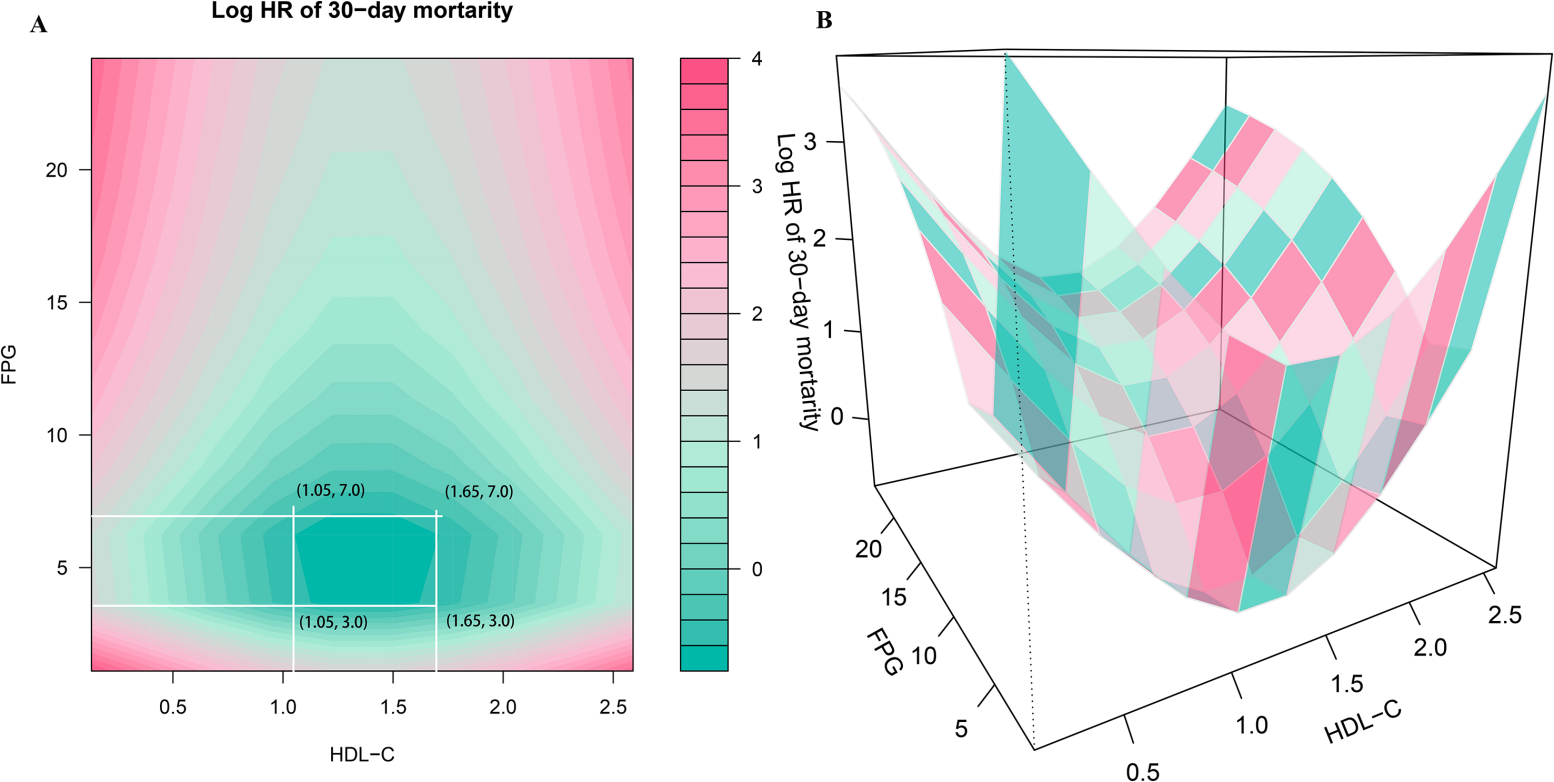
Two-dimensional heatmaps **(A)** and three-dimensional surface plots **(B)** of FPG, HDL-C levels, and 30-day all-cause mortality in ADHF patients. In the 3D surface plot **(B)**, it can be observed that the association of combined HDL-C and FPG assessment with mortality risk presents a concave pattern, with an obvious minimum point of mortality risk. In this heatmap **(A)**, the color gradient represents the combined effect of FPG and HDL-C levels on mortality risk, the deeper the blue, the stronger the negative association with mortality, while the deeper the red, the stronger the positive association with mortality. Using the ruler tool in Adobe Photoshop, we roughly measured in the heatmap that when HDL-C levels range from 1.05 to 1.65 mmol/L and FPG levels range from 3.0 to 7.0 mmol/L, the mortality risk falls within the deepest blue band, indicating the lowest mortality risk. FHR, fasting plasma glucose to high-density lipoprotein cholesterol ratio; ADHF, acute decompensated heart failure. Adjusted for gender, age, hypertension, diabetes, stroke, CHD, NYHA classification, dinking status, smoking status, LVEF, WBC, RBC, PLT, AST, GGT, ALB, Cr, UA, TC, TG, LDL-C, NT-proBNP.

Two-dimensional heatmaps and three-dimensional surface plots were employed to visualize the joint association patterns of FPG and HDL-C with 30-day mortality risk in ADHF patients (**Figure 4**). The results revealed a complex concave U-shaped association in their combined effect. This finding aligns with RCS analysis results, suggesting that metabolic homeostasis dysregulation may act as a critical mechanism underlying adverse outcomes in ADHF. Using the ruler tool in Adobe Photoshop, we roughly measured the numerical ranges in the heatmap, finding that when HDL-C levels ranged from 1.05 to 1.65 mmol/L and FPG levels ranged from 3.0 to 7.0 mmol/L, the mortality risk fell within the deepest blue band, indicating the lowest mortality risk.

### Subgroup analysis

We further evaluated the association between FHR and 30-day mortality in ADHF patients across subgroups stratified by age, gender, NYHA functional class, LVEF, and clinical comorbidities. The likelihood ratio test revealed that hypertension and CHD may be potential effect modifiers for the FHR-mortality association, with borderline significant interaction effects (**Table 3**; *P*-interaction for hypertension = 0.07, *P*-interaction for CHD = 0.08). Specifically, ADHF patients with comorbid hypertension or CHD exhibited a relatively higher FHR-associated 30-day mortality risk compared to those without these comorbidities.

**Table 3:** Stratified analysis showed the relationship between FHR and 30-day mortality in patients with ADHF of different ages, gender, NYHA classification, LVEF and whether combined with hypertension/diabetes/stroke/CHD.

Subgroup	HR (95%CI)	*P* for interaction
Age (years)		0.64
19-68	1.06 (1.00, 1.13)	
69-96	1.05 (1.01, 1.08)	
Gender		0.81
Male	1.06 (1.03, 1.09)	
Female	1.05 (1.01, 1.10)	
NYHA classification		0.80
III	1.05 (1.00, 1.09)	
IV	1.05 (1.02, 1.09)	
LVEF		0.35
< 50%	1.07 (1.02, 1.13)	
≥ 50%	1.04 (1.01, 1.08)	
Hypertension		0.07
Yes	1.08 (1.04, 1.12)	
No	1.03 (0.99, 1.07)	
Diabetes		0.60
Yes	1.06 (1.02, 1.10)	
No	1.05 (1.01, 1.08)	
Stroke		0.56
Yes	1.08 (1.01, 1.15)	
No	1.06 (1.02, 1.09)	
CHD		0.08
Yes	1.07 (1.04, 1.11)	
No	1.03 (0.99, 1.07)	

ADHF, acute decompensated heart failure; CHD, coronary heart disease; NYHA, New York Heart Association; LVEF, left ventricular ejection fraction; FHR, fasting plasma glucose to high-density lipoprotein cholesterol ratio.

Models adjusted for the same covariates as in model III (**Table 3**), except for the stratification variable.

### Predictive performance of FHR and its components for 30-day mortality in ADHF patients

**Table 4** presents the predictive performance of FHR and its components (FPG, HDL-C) for 30-day mortality in ADHF patients. The results demonstrated that FHR significantly outperformed both FPG and HDL-C alone in predicting 30-day mortality (All DeLong *P* < 0.05), with AUC values of 0.71, 0.62, and 0.65, respectively. Furthermore, the optimal cutoff value of FHR for predicting 30-day mortality was calculated as 6.72, with a sensitivity of 0.64 and specificity of 0.69.

**Table 4:** ROC analysis compares the predictive value of the FPG, HDL-C and the FHR for 30-day all-cause mortality in ADHF patients.

Variable	AUC	95%CI low	95%CI upp	Cut off value	Specificity	Sensitivity
FPG*	0.62	0.57	0.68	6.45	0.80	0.44
HDL-C*	0.65	0.60	0.69	0.91	0.62	0.61
FHR	0.71	0.66	0.75	6.72	0.69	0.64

AUC, area under the curve; FPG, fasting plasma glucose; HDL-C, high-density lipoprotein cholesterol ratio FHR, fasting plasma glucose to high-density lipoprotein cholesterol ratio.

*P < 0.001, compare with FHR.

### Exploratory mediation analysis

**Figure 5:**
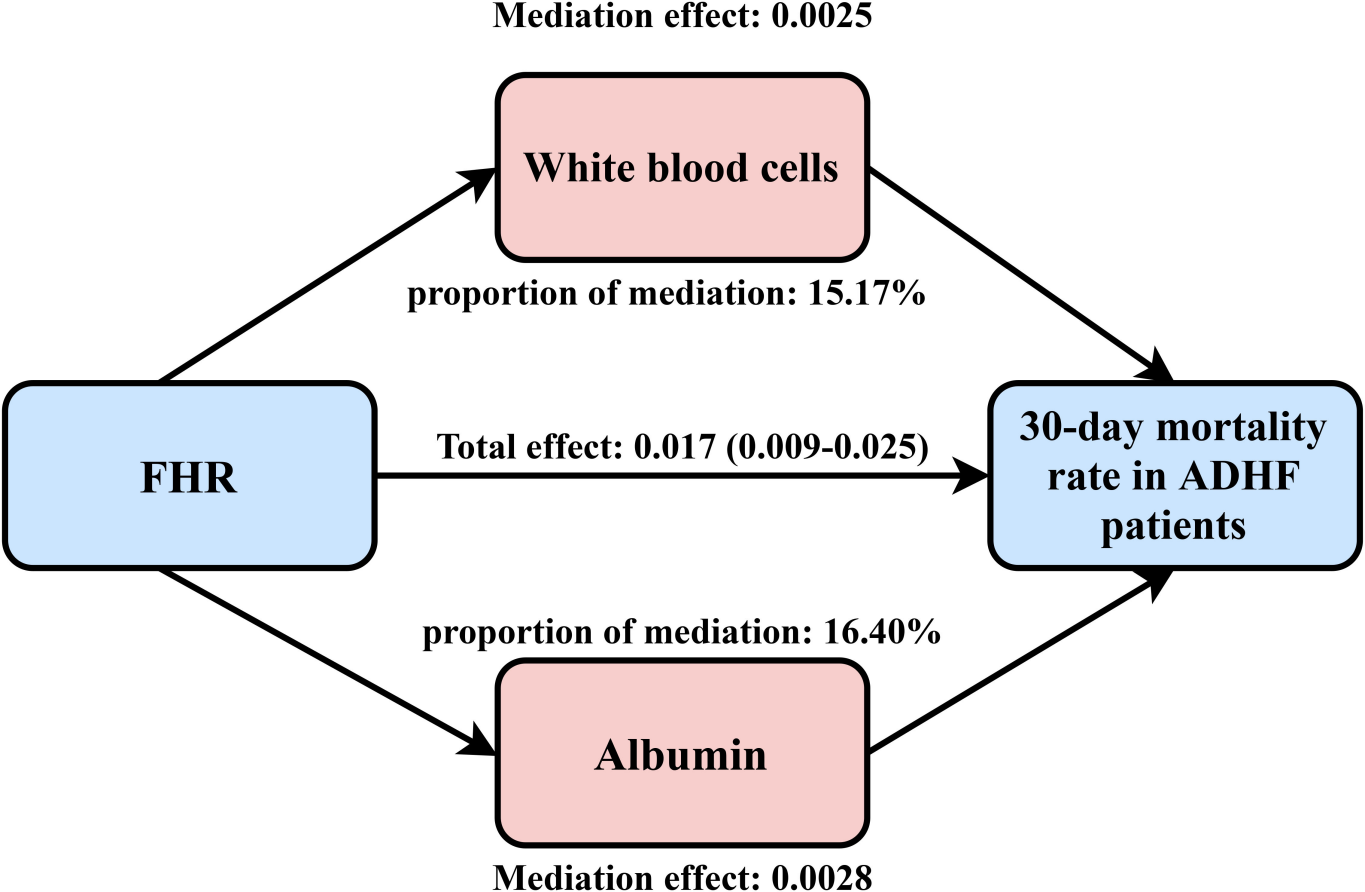
Path diagram for mediational model. FHR, fasting plasma glucose to high-density lipoprotein cholesterol ratio; ADHF, acute decompensated heart failure. Adjusted for gender, age, hypertension, diabetes, stroke, CHD, NYHA classification, dinking status, smoking status, LVEF, WBC, RBC, PLT, AST, GGT, ALB, Cr, UA, TC, TG, LDL-C, NT-proBNP.

An exploratory mediation analysis was conducted using a mediation model to investigate the potential mediating roles of the inflammatory factor WBC, oxidative stress marker GGT, and nutritional factor ALB in the association between FHR and 30-day mortality in ADHF patients (**Table 5**). The results demonstrated that, except for GGT, both WBC and ALB significantly mediated the FHR-mortality association. Specifically, WBC and ALB accounted for approximately 15.17% and 16.4% of the mediating effect, respectively (**Figure 5**).

**Table 5:** Mediated analysis was performed to explore the roles of inflammation, oxidative stress and nutritional pathways in the association between FHR and the 30-day mortality rate in ADHF patients.

Mediator	Total effect	Mediation effect	Direct effect	PM(%)	*P*-value of PM
WBC	0.017 (0.009, 0.025)	0.0025 (0.0012, 0.0046)	0.014 (0.006, 0.022)	15.17	<0.01
GGT	0.017 (0.009, 0.025)	0.0000 (-0.0003, 0.0003)	0.017 (0.010, 0.026)	0.02	0.97
ALB	0.017 (0.009, 0.025)	0.0028 (0.0011, 0.0044)	0.014 (0.007, 0.023)	16.40	<0.01

PM, proportion mediate; ADHF, acute decompensated heart failure; WBC, white blood cell count; GGT, gamma-glutamyl transferase; ALB, albumin.

The model adjusted for gender, age, hypertension, diabetes, stroke, CHD, NYHA classification, dinking status, smoking status, LVEF, RBC, PLT, AST, Cr, UA, TC, TG, LDL-C, NT-proBNP.

### Sensitivity analysis

In the validation cohort of U.S. patients with congestive HF, a positive association was observed between FHR and mortality prognosis (**Supplementary Table 2**). This finding suggests that FHR may be applicable for assessing adverse outcomes in multiethnic HF patients. After further adjusting the final model for treatment factors (including insulin, SGLT2 inhibitors, statins, or diuretics), the association remained consistent with the primary findings (**Supplementary Table 3**). Furthermore, after conducting further stratified analyses by medications, we observed no significant differences between subgroups (**Supplementary Table 4**, all *P*-interaction > 0.05). Finally, we also performed stratified analyses based on the clinical factor of BMI, and the results showed that the association between FHR and mortality did not differ significantly between obese and non-obese populations (**Supplementary Table 4**; *P*-interaction > 0.05).

## Discussion

This study, based on the China Jiangxi-ADHF II cohort, investigated the association between FHR and adverse outcomes in patients with ADHF, providing novel insights for risk stratification. Based on the current findings, we propose the establishment of a synergistic monitoring system for FPG and HDL-C to regulate metabolic homeostasis, thereby optimizing risk stratification in ADHF.

ADHF has emerged as one of the most challenging conditions to manage effectively due to its high recurrence risk and poor prognosis (29). Epidemiological studies indicate that ADHF patients face 30-day readmission and mortality rates as high as 25% and 10%, respectively (5–7). This poor prognosis is driven in part by metabolic disorders, characterized by disruption of glucose and lipid homeostasis, which promote ADHF progression through mechanisms such as inflammatory cascade activation, exacerbated oxidative stress, and aggravated IR, ultimately worsening clinical outcomes (13–15, 25, 30–35). Given the critical role of glucose and lipid metabolism in HF prognosis, this study uniquely developed the FHR ratio (FPG-to-HDL-C ratio) as a novel composite metabolic assessment metric. Current research on FHR primarily focuses on two domains: (1) Metabolic diseases: Yu et al., in a large-scale cross-sectional analysis of National Health and Nutrition Examination Survey data, reported that FHR was significantly associated with a 1.16-fold increased risk of non-alcoholic fatty liver disease, outperforming individual FPG or HDL-C indicators in predictive value (36); this finding was later validated by Jin et al. (37); (2) Cardiovascular diseases: Deng et al. observed that among patients with acute coronary syndrome, an FHR >3.02 was associated with a 12% increase in short-term risk of major adverse cardiovascular events. Similarly, an FHR >3.0 was linked to a 12% increase in short-term cardiovascular mortality risk (38). This study further confirms that FHR retains significant predictive value for short-term adverse outcomes in ADHF patients, extending its risk-stratification utility across different cardiovascular emergencies. Notably, we identified a U-shaped curve association between FHR and 30-day mortality in ADHF patients, with the nadir of risk corresponding to an FHR value of approximately 4–5. These findings suggest that in clinical practice, monitoring FHR levels can help identify high-risk ADHF patients. Aiming to maintain FHR within a range of 4–5 may be a useful prognostic strategy, rather than merely pursuing the “normalization” of FPG or HDL-C levels.

Another key finding of this study emerged from the two-dimensional heatmaps and three-dimensional surface plots. The analysis revealed that the 30-day mortality risk in ADHF patients was minimized when both FPG and HDL-C levels were maintained within specific ranges. Recent studies have revealed a U-shaped association between glucose levels and clinical outcomes in acute HF patients, with the lowest all-cause mortality observed when glucose levels were maintained between 5–7 mmol/L (15, 39–41). Similarly, these U-shaped or J-shaped associations have been observed in patients with baseline diabetes or critical illnesses: the risk of adverse clinical outcomes was minimized when glucose was controlled within 4–10 mmol/L (42–45). These findings are highly consistent with our current results. Notably, the pathological mechanisms linking hypoglycemia to adverse outcomes are multifactorial, potentially involving sympathetic-adrenal system activation, abnormal cardiac repolarization, vascular inflammation, and thrombosis (46, 47). Beyond glucose, the U-shaped association between HDL-C and adverse outcomes has also gained growing attention in recent studies. Historically, classical theory held that HDL-C exerts cardiovascular protective effects by mediating reverse cholesterol transport, and the notion that “higher HDL-C levels are better” was widely accepted among most researchers (48, 49). However, emerging evidence appears to be reshaping this paradigm. In a long-term follow-up study of HF patients, Abudouwayiti et al. identified a U-shaped association between HDL-C quartiles (Q1-Q4) and all-cause mortality, with the lowest mortality risk observed at HDL-C levels of 0.94–1.14 mmol/L (50). Additionally, Liu et al. demonstrated a U-shaped association in a cohort study of 19,945 patients with CHD from the United Kingdom Biobank and Emory Cardiovascular Biobank, where HDL-C levels of 1.04–1.55 mmol/L were associated with the lowest all-cause mortality risk (51). Similar findings have been reported in patients with hypertension and diabetes, where the risk of major adverse cardiovascular events was lowest when HDL-C levels were maintained between 40–80 mg/dL (52, 53). In the general population, a pooled analysis of 37 prospective cohort studies demonstrated a J-shaped association between HDL-C levels and all-cause mortality, with the lowest risk observed at HDL-C levels of 54–58 mg/dL (54). In summary, these studies across diverse patient populations suggest that compared to moderate HDL-C levels, higher HDL-C may increase adverse outcomes risk. The mechanisms underlying the association between elevated HDL-C and adverse outcomes remain incompletely understood, potentially involving rare genetic variants or functional dysregulation (55, 56). These findings highlight the need to revise the traditional “higher-is-better” paradigm for HDL-C management, establish disease-specific “optimal range” strategies, and promote a paradigm shift from quantity-based measurement to functional assessment in clinical practice.

It is noteworthy that the association between FHR and mortality risk in ADHF patients exhibited a borderline significant interaction in subgroups with hypertension and CHD. Specifically, ADHF patients with comorbid hypertension or CHD exhibited a relatively higher 30-day mortality risk associated with FHR. Based on previous relevant findings, we further analyzed the specificity of the association between FHR and mortality risk in these subgroups. First, regarding FHR’s components, patients with hypertension or CHD commonly exhibit glucose metabolic dysregulation (57, 58) and lipid metabolic disorders (59, 60), which likely elevate FHR values in these populations, thereby increasing mortality risk. Our current data support this hypothesis, demonstrating higher FHR levels in patients with hypertension (6.78 ± 3.97 vs. 6.48 ± 4.00 mmol/L, *P* = 0.065) or CHD (7.05 ± 4.19 vs. 6.41 ± 3.87 mmol/L, *P* < 0.001) compared to those without these comorbidities. Secondly, from the perspective of metabolic imbalance, hypertension serves as a core component of metabolic syndrome (12). The coexistence of hypertension with glucose and lipid metabolic disorders may generate synergistic effects that exacerbate IR, activate the renin-angiotensin-aldosterone system and sympathetic nervous system, and subsequently lead to structural and functional deterioration of the heart (61, 62), thereby worsening clinical outcomes in ADHF patients. The primary pathological feature of CHD is the accumulation of atherosclerotic plaques in epicardial coronary arteries, causing luminal stenosis or occlusion, which can induce myocardial ischemia, hypoxia, or necrosis (63). Glucose and lipid metabolic dysregulation, particularly elevated FPG and reduced HDL-C, further promotes atherosclerosis through multiple pathological mechanisms, thereby exacerbating myocardial ischemia, injury, and dysfunction (48, 49, 64), creating a vicious cycle of metabolic dysfunction-ischemia that worsens adverse outcomes in ADHF patients. Finally, from the perspective of synergistic exacerbation by underlying diseases: As two well-established major risk factors for HF development (1), both hypertension and CHD can independently drive HF progression (65, 66). When HF patients progress to the acute decompensated phase, comorbid hypertension or CHD exerts a synergistic effect, which may further amplify the mortality risk associated with elevated FHR. Evidence shows that ADHF patients with comorbid hypertension or CHD often experience worse clinical outcomes (66–68). Therefore, based on the aforementioned analyses, the distinct findings observed in hypertension or CHD subgroups may stem from a synergistic amplification effect between glucolipid metabolic dysregulation and these underlying diseases. These observations underscore the clinical importance of closely monitoring FHR in ADHF patients, particularly in those with comorbidities such as hypertension and CHD.

### Strengths and limitations

To our knowledge, this is the first study to evaluate the association between FHR and 30-day mortality in ADHF patients. The current findings provide valuable insights for optimizing monitoring strategies for metabolic factors in ADHF patients.

However, this study has several limitations. (1) As the study population was primarily derived from a Chinese cohort, caution is warranted when generalizing these findings to other ethnic groups. Although sensitivity analyses demonstrated that FHR is equally effective in assessing mortality prognosis in U.S. HF patients, this conclusion still requires further validation in more diverse prospective multiethnic cohorts. (2) Although we performed comprehensive adjustments for known confounders using robust statistical methods, the influence of unmeasured confounding factors cannot be completely excluded. (3) As a retrospective cohort analysis, this study has the following methodological limitations: i) The non-interventional study design restricts evaluation of treatment regimens and therapeutic efficacy in ADHF patients. ii) Although a significant association between FHR and prognosis was observed, the observational nature of the study precludes us from establishing causal relationships. (4) Repeated measurement data of FHR were unavailable in this study, limiting our ability to assess the dynamic changes of FHR and their prognostic impact on ADHF outcomes. Future studies should focus on the temporal evolution patterns of FHR. (5) Although 30-day follow-up can effectively assess the acute mortality risk of ADHF, it is difficult to capture the time-dependent effects of glycolipid metabolic disorders on medium- to long-term outcomes. Therefore, an extended follow-up is required to further investigate the predictive value of FHR for medium- to long-term prognosis in HF patients. (6) In the present study, the location of the “concave surface” in the heatmap and 3D surface plot was estimated only roughly using simple tools. Future technological advances will be necessary to enhance the quantification of regression analysis results in 3D space.

## Conclusion

This cohort study based on the population in Jiangxi, China, first uncovered a U-shaped association between FHR and 30-day mortality in ADHF patients, and identified the synergistic effects of FPG and HDL-C on mortality risk. Based on the current study findings, we propose a “metabolic synergistic management” strategy for ADHF patients from a clinical practice perspective: maintaining FPG levels between 3.0-7.0 mmol/L and HDL-C levels between 1.05-1.65 mmol/L. Additionally, enhancing the assessment of nutrition and inflammation in patients with an elevated FHR could significantly aid in disease management.

## Data Availability

The raw data supporting the conclusions of this article will be made available by the authors, without undue reservation.
