# Supplementary material for: Independent and joint associations of glucose modified by high-density lipoprotein cholesterol with mortality in heart failure patients: evidence from the Jiangxi, China cohort
Source: Front Endocrinol (Lausanne). 2025 Oct 29;16:1680746. doi: 10.3389/fendo.2025.1680746 (PMC12604991; doi:10.3389/fendo.2025.1680746)
Supplement: Supplementary file 1 [file DataSheet1.docx]

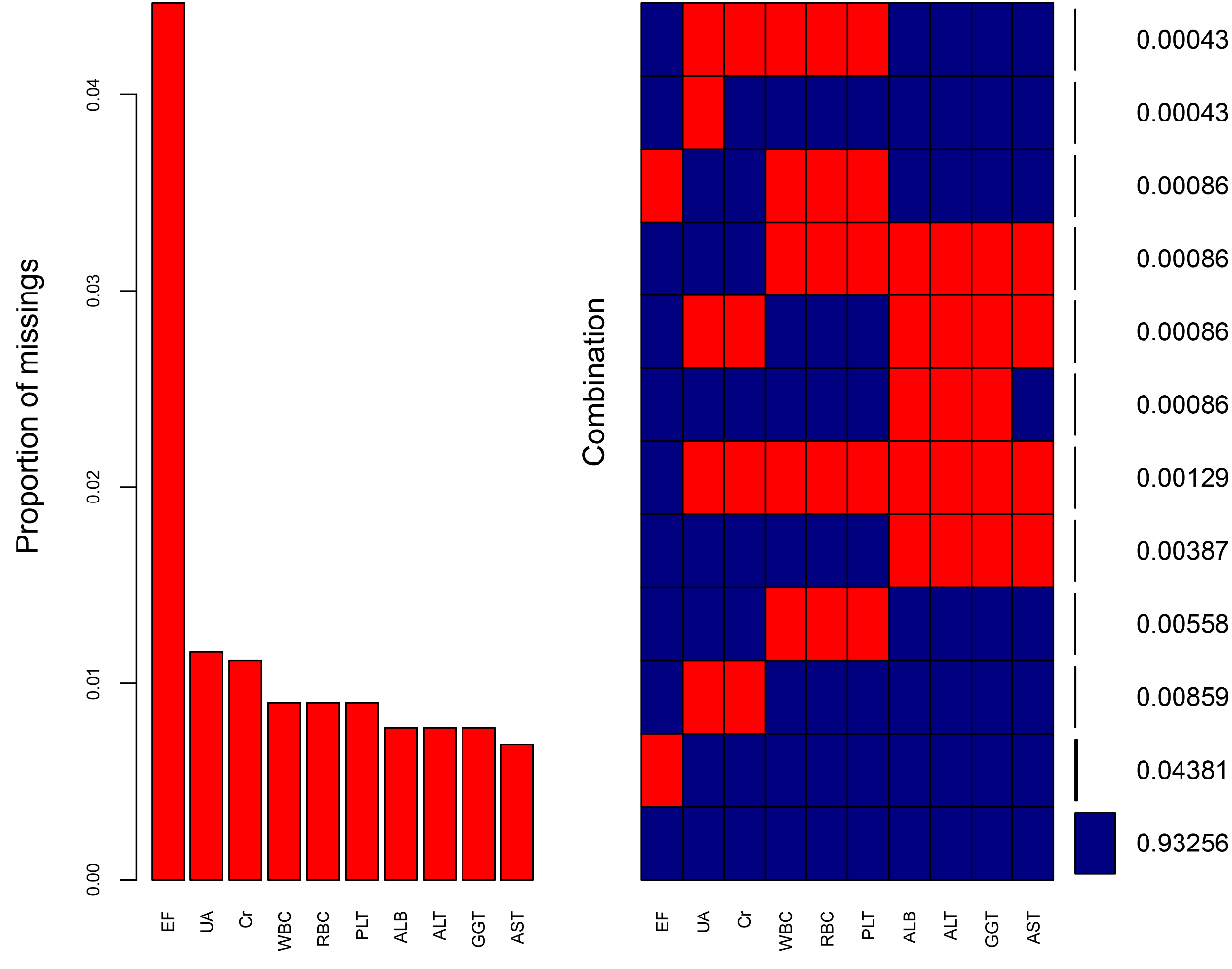


**Supplementary Figure 1:** Missing data cross-information diagram. LVEF: left ventricular ejection fraction; Cr: creatinine; ALT: alanine aminotransferase; AST: aspartate aminotransferase; GGT: gamma-glutamyl transferase; UA: uric acid; WBC: white blood cell count; RBC: red blood cell count; PLT: platelet count; ALB: albumin.
