# Supplementary material for: Independent and joint associations of glucose modified by high-density lipoprotein cholesterol with mortality in heart failure patients: evidence from the Jiangxi, China cohort
Source: Front Endocrinol (Lausanne). 2025 Oct 29;16:1680746. doi: 10.3389/fendo.2025.1680746 (PMC12604991; doi:10.3389/fendo.2025.1680746)
Supplement: Supplementary file 2 [file Table1.docx]

Supplementary Table 1. The missing number and rate of covariates.

| Variables | Non- Missing | Missing |
| --- | --- | --- |
| Gender | 2328 | 0 |
| Age | 2328 | 0 |
| Hypertension | 2328 | 0 |
| Diabetes | 2328 | 0 |
| Stroke | 2328 | 0 |
| CHD | 2328 | 0 |
| NYHA classification | 2328 | 0 |
| Smoking status | 2328 | 0 |
| Drinking status | 2328 | 0 |
| LVEF | 2224 | 104 |
| WBC | 2307 | 21 |
| RBC | 2307 | 21 |
| PLT | 2307 | 21 |
| ALB | 2310 | 18 |
| ALT | 2310 | 18 |
| AST | 2312 | 16 |
| GGT | 2310 | 18 |
| Cr | 2302 | 26 |
| UA | 2301 | 27 |
| TG | 2328 | 0 |
| TC | 2328 | 0 |
| LDL-C | 2328 | 0 |
| NT-proBNP | 2328 | 0 |
| FHR | 2328 | 0 |

Abbreviations as in Table 1.

Supplementary Table 2: Multivariable Cox regression analysis of the association between FHR and 30-day all-cause mortality in patients with congestive heart failure in the US Cohort.

|  | HR (95%CI) |
| --- | --- |
| FHR | 1.07 (1.02, 1.12) |
| FHR (quartiles) |  |
| Q1 | 1.0 |
| Q2 | 0.97 (0.65, 1.45) |
| Q3 | 0.78 (0.49, 1.23) |
| Q4 | 1.05 (0.61, 1.81) |
| *P*-trend | 0.99 (0.83, 1.18) |

Abbreviations: ADHF: acute decompensated heart failure; HR: hazard ratios; CI: Confidence interval; FHR: fasting plasma glucose to high-density lipoprotein cholesterol ratio.

Adjusted for: Gender, age, hypertension, diabetes, stroke, CHD, dinking status, smoking status, WBC, RBC, PLT, AST, GGT, Cr, UA, TC, TG, LDL-C, NT-proBNP.

Supplementary Table 3: Multivariable Cox regression analysis of the association between FHR and 30-day all-cause mortality in patients with ADHF.

|  | HR (95%CI) |
| --- | --- |
| FHR | 1.05 (1.02, 1.08) |
| FHR (quartiles) |  |
| Q1 | 1.0 |
| Q2 | 1.20 (0.60, 2.42) |
| Q3 | 1.94 (1.01, 3.73) |
| Q4 | 3.24 (1.63, 6.42) |
| *P*-trend | 1.52 (1.22, 1.89) |

Abbreviations: ADHF: acute decompensated heart failure; HR: hazard ratios; CI: Confidence interval; FHR: fasting plasma glucose to high-density lipoprotein cholesterol ratio.

Adjusted for: Gender, age, hypertension, diabetes, stroke, CHD, NYHA classification, dinking status, smoking status, LVEF, WBC, RBC, PLT, AST, GGT, ALB, Cr, UA, TC, TG, LDL-C, NT-proBNP, insulin therapy, SGLT2 inhibitor therapy, statin therapy and diuretic therapy.

Supplementary Table 4: Analyze the association between FHR and mortality in ADHF patients across BMI and medication treatment subgroups.

| Subgroup | HR (95%CI) | *P* for interaction |
| --- | --- | --- |
| BMI |  | 0.28 |
| <28 | 1.10 (1.04, 1.16) |  |
| ≥28 | 0.88 (0.54, 1.42) |  |
| Insulin therapy |  | 0.42 |
| Yes | 1.05 (1.02, 1.09) |  |
| No | 1.06 (0.99, 1.12) |  |
| SGLT2 inhibitor therapy |  | 0.33 |
| Yes | 1.07 (1.02, 1.15) |  |
| No | 1.05 (1.01, 1.08) |  |
| Statin therapy |  | 0.62 |
| Yes | 1.05 (1.01, 1.12) |  |
| No | 1.05 (0.99, 1.07) |  |
| Diuretic therapy |  | 0.52 |
| Yes | 1.05 (1.02, 1.09) |  |
| No | 1.04 (0.94, 1.11) |  |

Abbreviations: ADHF: acute decompensated heart failure; HR: hazard ratios; CI: Confidence interval; FHR: fasting plasma glucose to high-density lipoprotein cholesterol ratio; SGLT2: selective sodium glucose cotransporter 2; BMI: body mass index.

Note: Models adjusted for the same covariates as in model III (Table 3), except for the stratification variable.
